# Short-Term Versus Long-Term Systemic Corticosteroid Use in the Acute Exacerbation of Chronic Obstructive Pulmonary Disease Patients

**DOI:** 10.21315/mjms2021.28.1.8

**Published:** 2021-02-24

**Authors:** Samah Alshehri, Mai Alalawi, Abdulrahman Makeen, Ammar Jad, Ahmed Alhuwaysi, Mohammed Alageeli, Mohannad Alshibani

**Affiliations:** 1 Department of Pharmacy Practice, Faculty of Pharmacy, King Abdulaziz University, Jeddah, Saudi Arabia; 2 Faculty of Medicine, King Abdulaziz University, Jeddah, Saudi Arabia; 3 Faculty of Medicine, Ibn Sina National College for Medical Studies, Jeddah, Saudi Arabia

**Keywords:** COPD exacerbation, corticosteroids, re-admission, 30-day mortality, length of hospitalisation

## Abstract

**Background:**

The administration of systemic corticosteroids in chronic obstructive pulmonary disease (COPD) exacerbation is the first line of management. The duration of this administration, however, is not well established in clinical practice. The objective of this study is to compare the clinical outcomes between short-term and long-term corticosteroid use in the acute exacerbation of COPD patients.

**Methods:**

A single-centre, retrospective cohort study was conducted. From 2014 to 2018, all patients over 40 years old with COPD who were admitted to the hospital with a case of COPD exacerbation and received systemic corticosteroids at presentation were included. The subjects were divided into two groups according to the duration of systemic corticosteroid therapy. The primary outcome was hospital re-admission within 180 days. The secondary outcomes were 30 days mortality and length of hospitalisation. The two groups were compared using an independent sample *t*-test, a Chi-square test, and a Mann-Whitney U test, according to the data type.

**Results:**

Eighty patients met the inclusion criteria. A total of 52 (65%) patients completed long-term therapy, while 28 (35%) patients were on short-term treatment. A total of 15 (28.8%) patients reached the primary endpoint in the long-term treatment group versus 19 (67.9%) in the short-term treatment group (*P* = 0.001). The 30-day mortality was 4 (7.7%) and 0 (0%), respectively, and the median length of hospitalisation was 6.5 and 7.5 days in the long-term group and short-term group, respectively (*P* = 0.32, *P* = 0.88).

**Conclusion:**

Long-term corticosteroid use in the management of acute COPD exacerbation was significantly associated with fewer 180 days re-admission. The duration of corticosteroid use remains controversial, and further studies are recommended to assess the relationship between patient profile and adherence to therapy post-discharge with re-exacerbation.

## Introduction

Chronic obstructive pulmonary disease (COPD) is considered to be a complex, progressive life-threatening lung disease (1). It is characterised by chronic airflow obstruction that interferes with normal lung function, and it is not completely reversible (1). Acute COPD exacerbation happens when there is a deterioration of respiratory symptoms that necessitate additional therapy (1). COPD is associated with a high mortality rate; it is considered to be the fourth leading cause of death worldwide (2). By 2020, COPD is estimated to be the third leading cause of death worldwide (2). The administration of systemic corticosteroids, along with short-acting beta-2 agonists, is considered the first line of management in COPD exacerbation (3). Their rules have been established to reduce the frequency of exacerbations, minimise the urge of intubation, and lower the overall mortality rate (3, 4).

Previous studies have confirmed that corticosteroids are a cornerstone in the management of COPD exacerbation (3). Systemic corticosteroids have positive impacts on clinical outcomes, such as reducing re-admission rates within 180 days and accelerating the recovery of forced expiratory volume in the first second (FEV1) (1, 5). The duration of corticosteroid use in COPD exacerbation was studied in a randomised control trial (reduction in the use of corticosteroids in exacerbated COPD [REDUCE] trial), which was designed to compare the 5-day use (short-term) versus the 14-day use (long-term or conventional) (6). The study reported the non-inferiority of the short duration in terms of re-exacerbation within 6 months. On the contrary, short-term treatment significantly limited exposure to glucocorticoids and their adverse effects (6). The duration of systemic corticosteroid use is still not well established in clinical practice, and differences regarding administration were observed (7, 8).

The aim of this study is to investigate whether or not there are any differences in clinical outcomes between short-term versus long-term corticosteroid administration defined as re-admission within 180 days. The study also aims to compare the length of hospitalisation and mortality rate between the long-term group and the short-term group in treating acute exacerbated COPD.

## Methods

A retrospective cohort study was conducted at a tertiary academic medical centre in Jeddah, Saudi Arabia. Approval was obtained from the Institutional Review Board (IRB). The screened data were from 2014 to 2018. The criteria for eligibility were all patients over 40 years old with a primary diagnosis of acute COPD exacerbation based on the Global Initiative for Chronic Obstructive Lung Disease (GOLD) diagnostic criteria who were admitted to hospital as a case of COPD exacerbation. In addition, the patients also had to be administered systemic corticosteroids at presentation. The exclusion criteria were patients with a history of asthma, pneumonia, home use of systemic corticosteroids prior to admission and unavailability of a treatment plan.

Data was extracted from electronic medical records using a standardised collection sheet that included demographics, comorbidities such as hypertension, diabetes, cardiovascular disease, endocrine disease, kidney disease, hepatic disease, smoking history and all data related to corticosteroid therapy (name, route, dose and duration). Patient characteristics during hospitalisation were also identified, including ICU admission, the requirement of mechanical ventilation and the length of hospitalisation. The primary outcome was the next exacerbation within 180 days. The secondary outcomes were mortality within 30 days and length of current hospitalisation. Baseline characteristics were described using measures of central tendency. The frequencies were presented as numbers (percentages). The two groups (short duration and long duration) were compared using an independent sample *t*-test, a Chi-square test, a Mann-Whitney U test and Fisher’s exact test, according to the data type. A two-sided alpha level of less than 0.05 was considered to be statistically significant. SPSS software, version 21 (IBM, Chicago, IL, USA), was used for data analysis.

## Results

### Study Population

**Figure 1:**
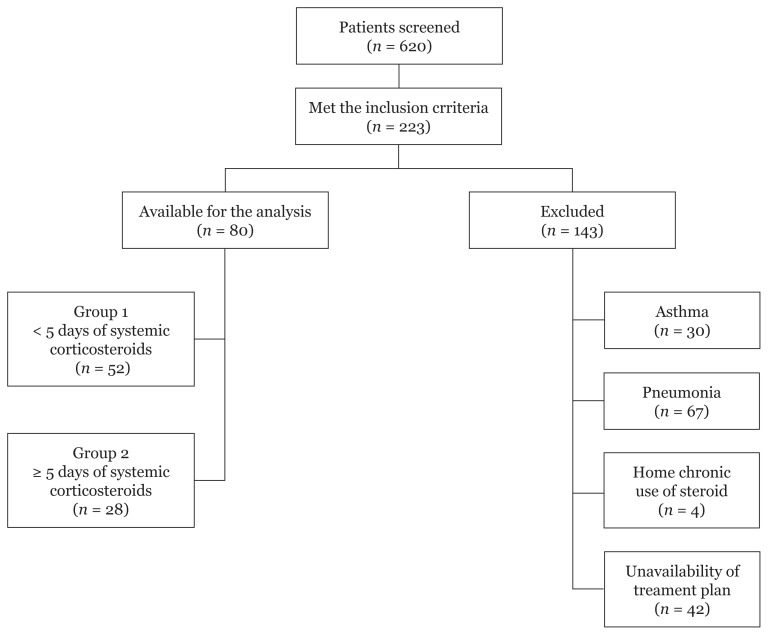
Flow of patients throughout the study

A total of 223 patients were evaluated for eligibility, of whom 80 patients met the inclusion criteria. The remaining 143 patients were excluded for various reasons. The majority of exclusions were patients who were diagnosed with community-acquired pneumonia and patients whose treatment plan was unavailable. Patients were divided into two groups based on the duration of corticosteroid therapy: long-term (conventional) duration and short-term duration. Long-term use is defined as > 5 days of systemic corticosteroids, while short-term use is defined as < 5 days of systemic corticosteroids (Figure 1).

### Baseline Characteristics

A total of 52 patients completed long-term (> 5 days) therapy (65%), while 28 were on short-term treatment (35%). The mean age was 66.2 and 68.3 in the long-term and short-term groups, respectively (*P* = 0.32*)*. Regarding the sex of patients, 29 (55.8%) in the long-term group and 18 (64.3%) in the short-term group were male (*P* = 0.46). Saudi nationality accounted for 23 (44.2%) and 15 (53.6%) in the long-term and short-term groups, respectively (*P* = 0.43). The most common comorbidities in our population were hypertension 10 (19.2%) versus 10 (35.7%), and diabetes 11 (21.2%) versus 4 (14.3%) in the long-term and short-term groups, respectively. Other comorbidities in the short-term group included one patient with coronary artery disease, one patient with hypothyroidism and one patient with hyperthyroidism. One patient had heart failure in the long-term group. Smoking status was divided into two categories: ‘ever smoke,’ which accounted for 38 (73.0%) versus 22 (78.6%) and ‘never smoke,’ which accounted for 6 (11.5%) versus 2 (7.1%) in the long-term group and short-term group, respectively. Twelve patients (23.1%) in the long-term group and 5 patients (17.9%) in the short-term were admitted to the intensive care unit (ICU) (*P* = 0.59). In addition, 11 patients (21.2%) were on mechanical ventilation in the long-term group versus 2 patients (7.1%) in the short-term group (*P* = 0.11). The baseline characteristics are illustrated in Table 1.

### Endpoints and Outcomes

A total of 15 patients (28.8%) were re-admitted within 180 days in the long-term treatment group in comparison to 19 patients (67.9%) in the short-term treatment group (*P* = 0.001) (Table 2).

The 30-day mortality rates were 4 (7.7%) and 0 (0%) in the long-term group and short-term group, respectively (*P* = 0.13). The median length of hospitalisation was 6.5 and 7.5 days in the long-term group and short-term group, respectively (*P* = 0.88).

## Discussion

We aimed to assess the use of corticosteroids in COPD exacerbation in a tertiary medical centre. Although GOLD guidelines state a clear recommendation of 5–7 days of treatment (1), the duration is still not well established in clinical practice, and differences regarding corticosteroid administration have been observed in previous studies (7, 8), as well as in our study.

Our findings indicate a significant difference between the two treatment groups in terms of re-admission within 180 days, where more re-exacerbations occurred in the short-term duration group (*P* = 0.001) compared to the long-term duration group. Our primary outcome was not consistent with the REDUCE randomised control trial that concluded the non-inferiority (hazard ratio = 0.95; 90% CI: 0.70–1.29) of short-term systemic corticosteroids as well as their advantage in decreasing the side effects related to corticosteroid exposure (6). Several explanations precipitate with the inconsistency of our primary outcome versus the REDUCE trial. The latter took place in Switzerland, as patients were screened for eligibility in five Swiss hospitals (6). The differing characteristics of our population, including adherence to medications and genetic factors, could be a reason why more re-exacerbations occurred in the short-term group. In addition, not all health care providers follow the updated guidelines for COPD management (9).

Our study population fell into the elderly category, where the mean age was 66.95 ± 10.25 years old. The elderly are well known for their poor adherence to and non-compliance with medications (10). Adherence to medications is a well-established factor that reduces the risk of recurrent exacerbation and improves the utilisation of different health care resources in COPD patients (11–13). However, especially in the Middle East, poor adherence to chronic medications has been documented for many diseases, including COPD (14). In addition, a cross-sectional study conducted in Saudi Arabia and Turkey that included 405 patients reported a high non-adherence percentage (49.2%) in COPD patients. Approximately half of the patients had at least one episode of exacerbation (52.5%) in the last year, with a higher ratio of exacerbations among Saudis (15). Besides considering the duration of corticosteroids received during hospitalisation, adherence can be an important factor affecting the number of re-admissions that occurred in each group as well. Patient adherence was neither assessed in the REDUCE trial nor in our study. However, based on the literature, non-adherence has a direct impact on the increasing number of hospital admissions and mortality rates (16). A diversity of factors lay beyond poor adherence to therapy (10, 17) that are recommended to be assessed prospectively in further studies among COPD patients in Saudi Arabia.

Another outcome of our study was 30-day mortality, which was insignificant between the two treatment groups (*P* = 0.132). Only four deaths occurred in the long-term group versus no deaths in the short-term group. Such findings were consistent with those reported in the REDUCE trial (hazard ratio = 0.93; 95% CI: 0.40–2.20; *P* = 0.87) (6). Previous studies have revealed that a greater frequency of exacerbations is associated with an increased risk of death (18, 19). In addition, early re-admission after a recent discharge — particularly within 30 days — is positively associated with a higher risk of death (20). Two patients out of the four who died were re-admitted within 180 days as a case of re-exacerbation, which reflects the severity of the cases and the positive association of early re-admission and mortality.

We acknowledge some limitations in our study. The sample size was small, especially in the short-term group. Disease severity during hospitalisation was not measured, including FEV1 values, due to the lack of documentation. As this is the nature of a retrospective design, GOLD classification was not evaluated during admission. Nevertheless, previous studies have illustrated that FEV1 values between the two groups are similar (6, 21).

## Conclusion

Long-term corticosteroid use in the management of acute COPD exacerbation was significantly associated with lower re-admission (180 days). The duration of corticosteroid use remains a controversy, and real clinical practice is different than the guideline recommendations. Further studies are recommended to assess patient profiles and adherence to therapy post-discharge and to establish whether these factors relate to re-admission despite corticosteroid duration.

## Figures and Tables

**Table 1:** Baseline characteristics

Characteristic	> 5 days (*n* = 52)	≤ 5 days (*n* = 28)	*P-*value*	All patients (*n* = 80)
Age, years (mean ± SD)*	66.2 ± 1.6	68.3 ± 1.3	0.32	67.0 ± 10.3
Sex, *n* (%)				
Male	29 (55.8)	18 (64.3)	0.46	47 (58.8)
Female	23 (44.2)	10 (35.7)		33 (41.3)
Nationality, *n* (%)				
Saudi	23 (44.2)	15 (53.6)	0.43	38 (47.5)
Non-Saudi	29 (55.8)	13 (46.4)		42 (52.5)
Comorbidity, *n* (%)	22 (42.3)	15 (53.6)	0.34	37 (46.3)
DM, *n* (%)	11 (21.2)	4 (14.3)	0.56	15 (18.8)
HTN, *n* (%)	10 (19.2)	10 (35.7)	0.10	20 (25)
Other, *n* (%)	3 (5.8)	1 (3.6)	1.00	4 (5)
Smoking status, *n* (%)				
Ever smoke	38 (73.0)	22 (78.6)	0.80	60 (79.9)
Never smoke	6 (11.5)	2 (7.1)		8 (10)
Not documented	8 (15.4)	4 (14.3)		12 (15)
ICU admission, *n* (%)	12 (23.1)	5 (17.9)	0.59	17 (21.3)
Mechanical ventilation, *n* (%)	11 (21.2)	2 (7.1)	0.13	13 (16.3)

Notes:

* *P*-values were calculated using Chi-square test for sex, nationality, comorbidities, smoking status, and ICU admission, independent sample *t*-test was used for age, Fisher’s exact test was used for diabetes, other comorbidities, and mechanical ventilation;

SD = standard deviation; *n* = number; DM = diabetes mellitus; HTN = hypertension; ICU = intensive care unit

**Table 2:** The outcomes of two groups of patients based on the duration of treatment

Outcome	> 5 days (*n* = 52)	≤ 5 days (*n* = 28)	*P*-value*	All patients
Re-admission within 180 days, *n* (%)	15 (28.8)	19 (67.9)	0.001	34 (42.5)
Mortality within 30 days, *n* (%)	4 (7.7)	0(0)	0.29	4 (5)
Length of hospitalisation, days median [IQR]	6.5 [9]	7.5 [7]	0.88	7 [9]

Notes:

* *P*-values were calculated using Chi-square for re-admission within 180 days; Mann-Whitney U test was used for length of hospitalisation; Fisher’s exact test was used for mortality within 30 days; *n* = number; IQR = interquartile range
